# Hydrolytic potential of five fungal supernatants to enhance a commercial enzyme cocktail

**DOI:** 10.1007/s10529-017-2371-9

**Published:** 2017-06-01

**Authors:** Ausra Peciulyte, Maria Pisano, Ronald P. de Vries, Lisbeth Olsson

**Affiliations:** 1 Department of Biology and Biological Engineering, Chalmers University of Technology, 412 96 Gothenburg, Sweden; 2 Department of Biotechnology and Biopharmaceutical Biosciences, University of Bari, 70125 Bari, Italy; 3 Fungal Physiology, CBS-KNAW Fungal Biodiversity Center & Fungal Molecular Physiology, Utrecht University, Utrecht, 3584 CT The Netherlands; 4 Wallenberg Wood Science Center, Chalmers University of Technology, 412 96 Gothenburg, Sweden

**Keywords:** *Aspergillus niger*, Celluclast 1.5L, Cellulose, Saccharification, *Trichoderma reesei*, Wheat bran

## Abstract

**Objectives:**

To evaluate the potential of enzyme cocktails produced by five filamentous fungi to supplement the industrial cellulase cocktail, Celluclast 1.5L, in order to improve the efficiency of saccharification.

**Results:**

The fungi were cultivated on wheat bran and the resulting supernatants were combined with Celluclast in enzymatic hydrolysis experiments to test their ability to hydrolyze wheat bran and five cellulose-rich substrates. The supernatant showing the best performance was that from an *Aspergillus niger* cellulase mutant. The addition of β-glucosidase only to the Celluclast cocktail was not as beneficial.

**Conclusion:**

Supplementing commercial cocktails with enzymes from carefully selected fungi may result in significantly more efficient saccharification of lignocellulosic materials. Furthermore, such an approach could lead to the identification of novel enzyme activities crucial for saccharification.

## Introduction

Plant polysaccharides are of considerable interest in many industrial applications, such as the production of biofuels, pulp, and paper, as well as for food and animal feed. However, plant polysaccharides are not easily accessible as they are composed of closely interlinked cellulose, hemicellulose and lignin, which are recalcitrant to degradation. Filamentous fungi are among the most potent producers of enzymes with the activities needed to disassemble these complex compounds in order to release the monosaccharides that serve as carbon and energy sources for fermentation. The environment in which the fungi are cultured will influence the amount and types of enzymes produced for the hydrolysis of plant cell walls (van den Brink and de Vries 2011).

The canonical enzymatic cellulose hydrolysis scheme involves cellobiohydrolases (CBHs), which processively degrade cellulose chains from the ends producing cellobiose, and endo-β-1,4-glucanases (EGLs), which introduce random cuts in the cellulose polymer, shortening polyglucan chains and generating reactive ends for the CBHs. β-Glucosidases (BGLs) hydrolyze soluble cellodextrins to glucose, mitigating product inhibition by CBHs and EGLs arising from cellobiose (Zhang and Lynd 2004). A recently discovered class of enzymes, lytic polysaccharide monooxygenases (LPMOs), which improve the efficiency of classic cellulases by introducing chain breaks in the polysaccharide chains using another (oxidative) mechanism, has changed our understanding of plant cell wall degradation (Johansen 2016). Cip proteins (Lehmann et al. 2015) and non-hydrolytic, expansin-like proteins, such as swollenin (Gourlay et al. 2015), have also been suggested to be relevant in the enzymatic hydrolysis of plant biomass. Construction and optimization of enzyme cocktails for improved saccharification is an active area of research (Andersen et al. 2008; Morrison et al. 2016). Many enzymatic activities are involved in the hydrolysis of cellulose, which is the simplest polysaccharide in plant cell walls having only one type of covalent bond, the β-(1,4)-d-glycosidic bond. The hydrolysis of cellulose is distinct from most other enzymatic reactions because it involves soluble enzymes acting on an insoluble substrate with a complex supramolecular structure (Peciulyte et al. 2015).

Emerging fungal genomes are revealing a large number of putative genes that may have potential in enzymatic hydrolysis. *Trichoderma reesei* (teleomorph *Hypocrea jecorina*) is the main industrial source of cellulases, and has the ability to produce and secrete large amounts of enzymes (Martinez et al. 2008). *T. reesei* is a poor producer of BGLs, and the saccharification of lignocellulosic materials therefore requires supplementation of *T. reesei* culture broth with enzymes from other sources. *Aspergillus niger*, one of the commonly used industrial *Aspergillus* species for the production of enzymes, has plant polysaccharide-degrading potential, but its efficiency in cellulose hydrolysis is limited (Culleton et al. 2013). An *A. niger* mutant showing reduced expression of the gene *noxR* from this fungus was produced by adaptive evolution, and found to have improved cellulase production (Patyshakuliyeva et al. 2016).

Identifying complementary enzymatic activities to existing ones is an area of great interest, and the ability of fungi other than the industrial enzyme producer *T. reesei* is being investigated. In this study, we explored the potential of five filamentous fungi to produce enzymes for cellulose hydrolysis using wheat bran as a carbon and energy source. The aim was to supplement the commercial enzyme cocktail Celluclast with fungal supernatants to evaluate whether the efficiency of the industrial cellulase cocktail could be enhanced.

## Materials and methods

### Enzymes and substrates

Celluclast 1.5L and Novozyme 188 enzyme cocktails were obtained from Novozymes A/S. Wheat bran purchased at a local store was used as the carbon and energy source for the cultivation of filamentous fungi. Enzymatic hydrolysis was performed with the enzymes produced on wheat bran and five cellulose-rich substrates: Avicel PH-101, and four types of fibers, never-dried (ND) and dried (D), produced from softwood. Avicel was purchased as powdered microcrystalline cellulose. The various fibers were produced from softwood biomass consisting of an industrially chipped and screened mixture of 40% Scots pine (*Pinus sylvestris*) and 60% Norway spruce (*Picea abies*) (thickness 2–8 mm). Chips with bark and knots were removed by hand. The chips were subjected to pre-hydrolysis followed by alkaline soda cooking (Karlström et al. 2014) to obtain Fibers 1(ND), which were delignified with oxygen to obtain Fibers 2(ND). Fibers 2(ND) were dried at 105 °C to obtain Fibers 2(D). Pre-hydrolyzed chips were subjected to kraft pulping followed by O_2_ delignification to obtain Fibers 3(ND). The ash content, carbohydrates and lignin content in the substrates were analyzed as described previously (Peciulyte et al. 2015) (Table 1). Fibers 2(D) were soaked in water, gently stirred and were then drained. The fibers were stored wet at 4 °C in airtight plastic bags until use.

**Table 1:** The chemical composition of the substrates analyzed (as % w/w)

Substrate	Avicel	Fibers 1(ND)	Fibers 2(ND) and Fibers 2(D)^a^	Fibers 3(ND)	Original biomass
Acid-insoluble lignin	nd	2.4	0.5	0.5	28.3
Acid-soluble lignin	nd	0.5	0.5	0.5	0.3
Extractives	nd	<0.3	<0.3	<0.3	2.8
Ash content	nd	<0.1	<0.1	<0.1	0.3
Xylose	2.2	1.5	1.5	1.7	6
Mannose	0.9	0.8	0.7	1.1	11.7
Arabinose	<0.1	<0.1	<0.1	<0.1	1.5
Galactose	<0.1	<0.1	<0.1	<0.1	2.4
Glucose	96.8	94.9	96.8	96.3	42.7
Total monosaccharides	99.9	97.2	99	99.1	64.3

*nd* not determined

^a^Carbohydrate composition was only analyzed in Fibers 2(ND). Drying of Fibers 2(ND) to produce Fibers 2(D) was not expected to change the chemical composition

### Strains, media and growth conditions

Spores of *T. reesei* (Rut C-30, NRRL 11460, ATCC 56765) were propagated on potato/dextrose/agar (PDA) plates at 30 °C for 6 days. The PDA medium consisted of 39 g PDA, 1 ml trace element solution, and deionized water to 1 l. The trace element solution was composed of 1 g ZnSO_4_·7H_2_O and 0.5 g CuSO_4_·5H_2_O in 100 ml Milli-Q water. *Trichoderma atroviride* (IMI 206040), an *A. niger* (CBS 140717) mutant (Patyshakuliyeva et al. 2016), *Podospora anserina* (FGSC #10383) and *Stagonospora nodorum* (CBS 438.87) were obtained from CBS. *T. atroviride* was cultivated on 15 g agar l^−1^ at 25 °C for 6 days. The *A. niger* mutant was cultivated on malt extract/agar (MEA) at 30 °C for 6 days. *P. anserina* and *S. nodorum* strains did not sporulate. Therefore, a small agar plug containing mycelium (1 mm diam.) was transferred from the edge of a vigorously growing colony on MEA to the center of a porous polycarbonate membrane (0.1 µm pore size, 76 mm diam. Osmonics, GE Water Technologies, Trevose, PA, USA), which was placed on a new agar plate with MEA and grown for 10 days at 25 °C. The fungal mycelia cannot penetrate the membrane. However, extracellular proteins can pass through the membrane and the fungus can utilize the nutrients from MEA.

Growth experiments were performed in liquid medium in 250 ml baffled shake-flasks, containing 50 ml cultivation medium. The medium was inoculated with sporulating strains by adding 10^6^ spores ml^−1^ suspended in the culture medium. Mycelia of non-sporulating strains were harvested and cut into small pieces prior inoculation of cultivation medium. *T. reesei* Rut C-30 and *T. atroviride* cultivation medium was composed of (per l): 4 g KH_2_PO_4_, 13.6 g (NH_4_)_2_SO_4_, 0.8 g CaCl_2_·2H_2_O, 0.6 g MgSO_4_·7H_2_O, 10 mg FeSO_4_·7H_2_O, 3.2 mg MnSO_4_·H_2_O, 2.8 mg ZnSO_4_·7H_2_O, 4 mg CoCl_2_·6H_2_O, 0.1 g Bacto peptone, at pH 4.5. The medium used to cultivate the *A. niger* mutant and *S. nodorum* was composed of (per l), 6 g NaNO_3_, 1.5 g KH_2_PO_4_, 0.5 g KCl, 0.5 g MgSO_4_, and 0.2 ml trace element solution (10 g EDTA, 4.4 g ZnSO_4_·7H_2_O, 1.0 g MnCl_2_·4H_2_O, 0.32 g CoCl_2_·6H_2_O, 0.32 g CuSO_4_·5H_2_O, 0.22 g (NH_4_)_6_Mo_7_O_24_·4H_2_O, 1.47 g CaCl_2_·2H_2_O and 1 g FeSO_4_·7H_2_O), at pH 6. The medium used to cultivate *P. anserina* was composed of (per l) 0.25 g KH_2_PO_4_, 0.3 g K_2_HPO_4_, 0.25 g MgSO_4_·7H_2_O, 0.5 g urea, 2.5 mg citric acid, 2.5 mg ZnSO_4_, 0.5 mg CuSO_4_, 125 µg MnSO_4_, 25 µg boric acid, 29 µg Na_2_MoO_4_·2H_2_O, 25 µg NH_4_Fe(SO_4_)_2_·12H_2_O, and 5 g dextrin, at pH 7. Each cultivation medium was supplemented with 2 ml L^−1^ vitamin solution consisting of (per l) 0.1 g thiamine, 1 g riboflavin 5-phosphate, 0.1 g *p*-aminobenzoic acid, 1 g nicotinamide, 0.5 g pyridoxine·HCl, 0.1 g pantothenic acid, and 0.02 g biotin. The cultivation media were supplemented with 10 g l^−1^ wheat bran as the carbon and energy source. *T. reesei* Rut C-30 and the *A. niger* mutant strain were cultivated at 30 °C, while the other strains were cultivated at 25 °C. The *A. niger* mutant strain was cultivated on a rotary shaker at 250 rpm and the other strains at 200 rpm. Each strain was cultivated in duplicate. After 6 days of cultivation, the fungal supernatants were harvested by centrifugation at 10,000×*g* for 10 min and stored at −20 °C until use.

### Protein quantification

The protein concentration in the fungal supernatants was assayed with the Pierce BCA protein assay kit according to the manufacturer’s instructions for microplate assay. Bovine serum albumin (BSA) was used as a standard. The same fungal growth medium as was used for cultivating the filamentous fungi was used as a diluent for the supernatants and BSA.

### Enzymatic activity measurements

All enzyme activity assays were performed in 50 mM sodium citrate buffer, at pH 4.8, in flat-bottomed 96-well plates. The filter paper activity (FPA) was measured employing 60 µl format, as described elsewhere (Xiao et al. 2004). One filter paper unit was defined as the amount of enzyme releasing 1 µmol reducing sugar from Whatman filter paper grade No. 1 per min. EGL activity was measured by incubating 30 µl enzyme solution with 30 µl 2% carboxymethyl cellulose (CMC) for 30 min at 50 °C, followed by the addition of 180 µl dinitrosalicylic acid reagent. The plate was sealed and incubated for 5 min at 95 °C. A reaction aliquot of 50 µl was transferred to a new plate, mixed with 250 µl deionized water and the absorbance was measured at 540 nm. One unit was defined as the amount of enzyme releasing 1 µmol reducing sugar from CMC per min. Glucose was used to obtain the standard curve for FPA and EGL activity measurements. For BGL activity measurements, 15 µl of several dilutions of fungal supernatant samples and pure BGL (EC 3.2.1.21) from *A. niger* (E-BGLUC, Megazyme International Ireland Ltd., Wicklow, Ireland) was incubated with 150 µl 5 mM 4-nitrophenyl-β-d-glucopyranoside (N7006, Sigma-Aldrich) as a substrate for 10 min at 50 °C. The reaction was stopped by adding 135 µl 0.5 M glycine/2 mM EDTA, at pH 10. The absorbance was measured at 405 nm. The final BGL activity was calculated as the average of three or four samples of the dilutions from the linear reaction range. One unit was defined as the amount of enzyme that produced 1 µmol 4-nitrophenol per min.

### Enzymatic hydrolysis

Screening of the enzymatic hydrolysis performance of the five filamentous fungi was performed in 96-well flat-bottomed plates for 24 h, at 100 rpm on a rotary shaker, at 50 °C in 50 mM sodium acetate buffer, pH 4.8. The final hydrolysis reaction volume was 280 µl. Experiments to evaluate the enzymatic hydrolysis performance of *A. niger* mutant supernatant were performed in 2 ml Eppendorf tubes on an adjustable angle mixing rotator. The reactants were scaled up to 2 ml keeping the same concentrations as in the experiment performed in 96-well plates. In the experiments where only BGL was used, the BGL activity loaded was 0.22 U ml^−1^, which corresponds to the BGL activity measured in the *A. niger* supernatant.

### Quantification of reducing sugars

The 60 µl format DNS assay (Xiao et al. 2004) without addition of filter paper was adapted for the quantification of reducing sugars. Glucose was used to obtain a standard curve.

### Analysis of monosaccharides in samples after enzymatic hydrolysis

The monosaccharides released by enzymatic hydrolysis were analyzed using a high-performance anion-exchange chromatography system coupled with a pulsed amperometric detector (HPAEC-PAD) (Dionex ISC-3000, Sunnyvale, CA, USA). A CarboPac PA1 analytical column (250 mm × 4 mm) and a guard column (50 mm × 4 mm) were used. Prior to analysis, the samples were boiled for 10 min to denature proteins, filtered through a 0.2 µm nylon filter, and the samples were diluted with Milli-Q water. Water was used as eluent at 1 ml min^−1^, and 300 mM NaOH was added at 0.5 ml min^−1^ before the detector. The column was cleaned with 200 mM NaOH dissolved in 170 mM sodium acetate. Sample injection was 25 µl, and the column was maintained at 30 °C during analysis. Calculations were performed using Chromeleon version 6.8 (Dionex).

## Results and discussion

### Protein production on wheat bran

Five filamentous fungi, *T. reesei*, *T. atroviride*, an *A. niger* cellulase mutant, *P. anserina* and *S. nodorum*, were used for enzyme production in the current study. They were selected based on their good growth on cellulosic and lignocellulosic substrates (data not shown) and the availability of their genome sequences. Wheat bran was chosen as the carbon and energy source for the growth of all fungi as it is a natural and complex nutrient-rich substrate that induces the expression of a broad range of genes encoding enzymes that act on plant cell walls. To assess protein production and concomitant enzyme activities, we measured protein concentrations and enzyme activities, such as total cellulase (FPase), EGLs (CMCase) and BGLs, in the supernatants on day six of fungal cultures (Table 2). The results were compared to the commercial enzyme cocktails, Celluclast and Novozyme 188, and, Cellic CTec2 (Table 2). The measurements were performed on the culture supernatants harvested by centrifugation, and possible losses of enzymes due to adsorption to the wheat bran, which contains a significant amount of lignin, were not considered. The highest protein concentration (1.5 mg ml^−1^) was in *T. reesei* culture supernatant, which also showed the highest FPase (0.2 U ml^−1^) and CMCase (2.6 U ml^−1^) activities. The highest BGL activity (5.2 U ml^−1^) was in the supernatant from the *A. niger* mutant. FPase activity was not detectable in this strain and the specific EGL activity was six times lower than in the supernatant from *T. reesei*.

**Table 2:** Protein titers and specific enzyme activities determined in the fungal supernatants after 6 days’ cultivation on wheat bran

Strain	Protein concentration (mg ml^−1^)	FPase (U mg^−1^)	CMCase (U mg^−1^)	BGL (U mg^−1^)
*T. reesei* Rut C-30	1.5 ± 0.3	0.2 ± 0.1	2.6 ± 0	0.1 ± 0
*T. atroviride*	0.7 ± 0	b.d.	1.4 ± 0.4	0.2 ± 0
*A. niger* mutant	0.4 ± 0.1	b.d.	0.2 ± 0.0	5.2 ± 0.2
*S. nodorum*	0.1 ± 0	b.d.	0.4 ± 0.2	0.8 ± 0
*P. anserina*	0.4 ± 0	b.d.	0.1 ± 0.1	b.d.
Celluclast 1.5L	127^a^	0.5	nd	0.1
Novozyme 188	220^a^	n.d.	nd	1.1
Cellic CTec2	161^a^	0.7	nd	0.02

Mean values and standard deviations of two replicates are presented

*b.d.* below the detection limit, *nd* not determined

^a^Determined as mg protein g^−1^ solution (Cannella and Jørgensen 2014)

The genomes of *A. niger* and *T. reesei* contain 15 (Pel et al. 2007) and seven BGLs, respectively (five of which are putative) (Foreman et al. 2003), which explains why more BGL activity was found in *A. niger* supernatant than in *T. reesei*. However, it was interesting that no FPase or EGL activities were found in the *A. niger* supernatant, despite its improved production of cellulases (Patyshakuliyeva et al. 2016). Expression of *noxR* was reduced by about 33 times in the *A. niger* mutant strain compared to its parental strain. *noxR* is a homolog of the Nox regulator, which has been identified in *P. anserina* (*PaNoxR*), and it has been shown to negatively affect cellulase production, indicating that cellulase production levels may be increased in a strain with down-regulated *noxR* (Brun et al. 2009).

### Enzymatic hydrolysis of wheat bran and cellulose-rich substrates

**Fig. 1:**
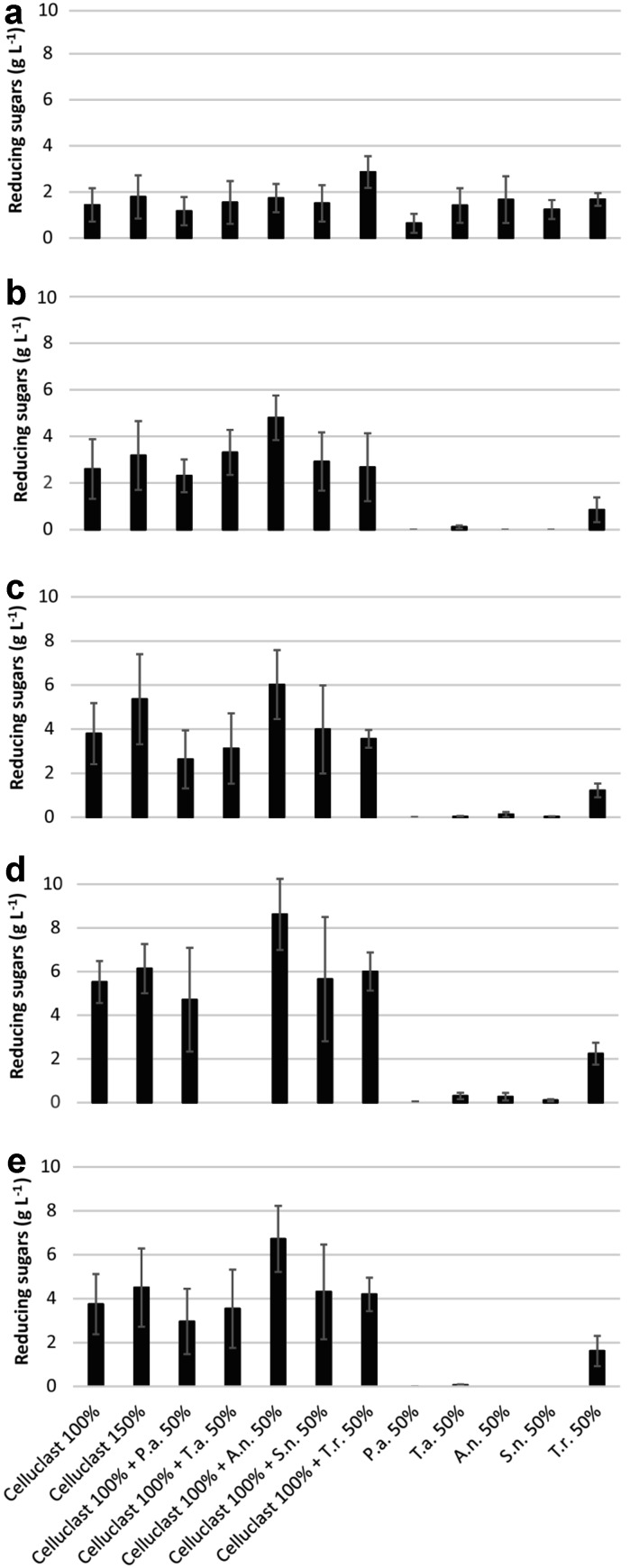
Reducing sugars (g l^−1^) measured by the DNS assay after 24 h hydrolysis at 50 °C of: **a** wheat bran, **b** Avicel, **c** Fibers 1(ND), **d** Fibers 2(ND), and **e** Fibers 2(D). 1% of carbohydrates loading (w/v) was used in the enzymatic hydrolysis reactions. Hydrolysis was performed on the following combinations of Celluclast and fungal supernatants: 9 mg protein of Celluclast (represented as 100%), 13 mg protein of Celluclast (represented as 150%), 9 mg protein of Celluclast and 4.5 mg protein of fungal supernatant (in total 150% protein), and 4.5 mg protein of fungal supernatant (represented as 50%) per g of carbohydrates. P.a. = *P. anserina*, T.a. = *T. atroviride*, A.n. = *A. niger*, S.n. = *S. nodorum* and T.r. = *T. reesei* Rut C-30. Mean values and standard deviations of six replicates are presented

Wheat bran and five cellulose-rich substrates were used in the enzymatic hydrolysis study. Enzymes were dosed based on protein concentration to ensure constant enzyme/substrate ratios in all the experiments. The separate fungal supernatants were each able to hydrolyze wheat bran to a similar extent as Celluclast (Fig. 1a). This indicates that the application of enzymes to the same substrate as was used for enzyme production may be advantageous, in agreement with some previous studies (Alvira et al. 2013), while no such advantage was observed in other studies (Jørgensen and Olsson 2006). Wheat bran was the substrate most recalcitrant to hydrolysis in this study (Fig. 1a). A plausible reason is the high lignin content, which could cause irreversible adsorption and steric hindrance to enzymes. Furthermore, the dry nature of the substrate has been shown to have negative effects on its hydrolysis (Peciulyte et al. 2015). When combined with Celluclast, *T. reesei* supernatant was the most efficient in the overall hydrolysis of wheat bran. The source of the enzymes in the Celluclast cocktail is *T. reesei*, but the culture medium used to produce it is proprietary information.

The ability of the supernatant of *T. reesei* to complement Celluclast shows that the production of cellulolytic enzymes is strongly dependent on the nature of the carbon source. One of the fungal supernatants evaluated for the hydrolysis of wheat bran was from *S. nodorum*, which is a pathogen of wheat (Hane et al. 2007). However, the hydrolysis of wheat bran with *S. nodorum* supernatant alone or in combination with Celluclast was not significantly better than any other of the fungal supernatants. When the fungal supernatants were loaded alone, without Celluclast, they all hydrolyzed wheat bran to a similar extent as Celluclast, except for the supernatant of *P. anserina*, which showed a lower hydrolysis yield than Celluclast alone. The five cellulose-rich substrates, namely Avicel and the four kinds of fibers, had similar chemical compositions, with a cellulose content ranging between 95 and 97% (Table 1), while the major difference between them was of structural nature (Peciulyte et al. 2015). The most recalcitrant cellulose-rich substrate was Avicel (Fig. 1b). Only *T. reesei* supernatant was able to hydrolyze the cellulose-rich substrates without the addition of Celluclast, but supplementation of Celluclast with *T. reesei* supernatant did not show an increase in enzymatic hydrolysis compared to Celluclast alone (Fig. 1b–e). The supernatant from the *A. niger* strain was best in the hydrolysis of Avicel, Fibers 2(ND) and Fibers 2(D) in combination with Celluclast (Fig. 1b, c, e).

Among the strains used in this study, *P. anserina* harbored the largest (105) (Espagne et al. 2008), *T. reesei* the lowest (16) and *A. niger* a moderate (55) number of carbohydrate-binding modules (CBMs) in their genomes. It is often suggested that the CBMs of cellulases are required for efficient saccharification of insoluble substrates. CBMs have been suggested to be an advantage for activity at low substrate loads, but a disadvantage for activity at high loads (Várnai et al. 2013). In the industrial saccharification process, it is desirable to use as high temperature and solids loadings as possible. The loading used in the present hydrolysis experiments was 1% (w/v) carbohydrates, which can be considered a low solids loading. If solids loading were the only important parameter in enzymatic hydrolysis, then enzymes with CBMs would be advantageous, suggesting that *P. anserina* could possibly be a good candidate for hydrolytic enzymes.

**Fig. 2:**
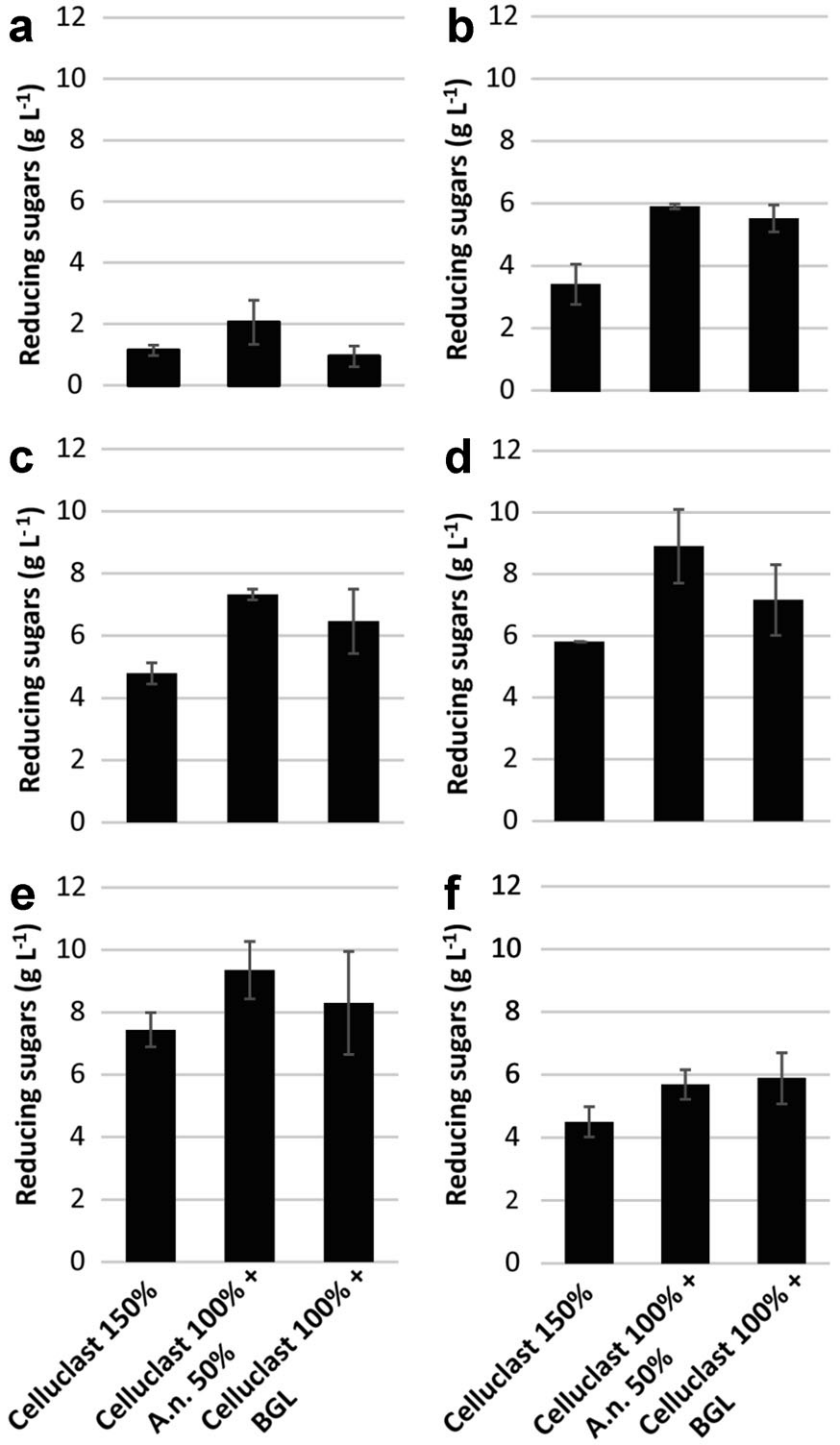
Concentrations of reducing sugars measured by the DNS assay after 24 h hydrolysis at 50 °C of: **a** wheat bran, **b** Avicel, **c** Fibers 1(ND), **d** Fibers 2(ND), **e** Fibers 3(ND) and **f** Fibers 2(D). Loading of the substrates, Celluclast and *A. niger* (A.n.) supernatant were the same as described in Fig. 1. Loading of pure β-glucosidase (BGL) was 0.22 U ml^−1^. Mean values and standard deviations of four replicates are presented

As many as 30, and 33, genes of the fungal LPMOs, auxiliary activity (AA9; previously known as GH61) family are present in *S. nodorum* and *P. anserina*, respectively, and three and seven are encoded by *T. reesei* and *A. niger*, respectively. LPMOs are known to oxidatively break down recalcitrant polysaccharide chains (Johansen 2016). However, the fungi with the highest number of AA9 genes did not show superior enzymatic hydrolysis of the cellulose-rich substrates. One plausible explanation of this is that the conditions during enzymatic hydrolysis of cellulose-rich substrates were not suitable for AA9 enzyme activity, as no reducing agent was added, which is required for the activity of these enzymes. Fibers 1(ND) and Fibers 2(ND) were produced in the same way, and differed only in that Fibers 1(ND) contained 3% residual lignin, while Fibers 2(ND) were delignified to 1% residual lignin. The slightly higher lignin content in Fibers 1(ND) thus reduced the enzymatic hydrolysis significantly (Figs. 1, 2). This suggests that the removal of lignin is important, even when only minor amounts are present, to improve the enzymatic hydrolysis of cellulose. The enzymatic hydrolysis yield from dried substrate (Fibers 2(D)) also showed a significant decrease compared to the never-dried substrate (Fibers 2(ND)), although the chemical composition of these fibers was the same, and drying changed only the structural properties (Figs. 1, 2).

### Possible reasons why the supernatant from the *A. niger* cellulase mutant enhanced the efficiency of the industrial enzyme mixture

The observation that a combination of *A. niger* supernatant and Celluclast gave the highest yield of reducing sugars (Fig. 1), led to the question of whether this was due to the higher BGL activity in the *A. niger* supernatant (Table 2), or synergistic effects between the enzymes in the supernatant and those present in Celluclast. To evaluate this, purified BGL from the *A. niger* strain was loaded together with Celluclast at the same activity as measured in the *A. niger* supernatant used for the enzymatic hydrolysis experiments (Fig. 2).

Supplementation of Celluclast with the *A. niger* supernatant resulted in a 1.8-times increase in the hydrolysis of wheat bran, while the addition of monocomponent BGL had no significant effect on the hydrolysis yield of wheat bran compared to Celluclast alone (Fig. 2a). However, the hydrolysis yield on all the cellulose-rich substrates was increased when Celluclast was supplemented with BGL; the greatest effect being seen on Avicel (1.6 times) (Fig. 2b–f). The supplementation of Celluclast with the *A. niger* supernatant resulted in better hydrolysis of the cellulose-rich substrates than with monocomponent BGL, except for Fibers 2(D) (Fig. 2).

**Fig. 3:**
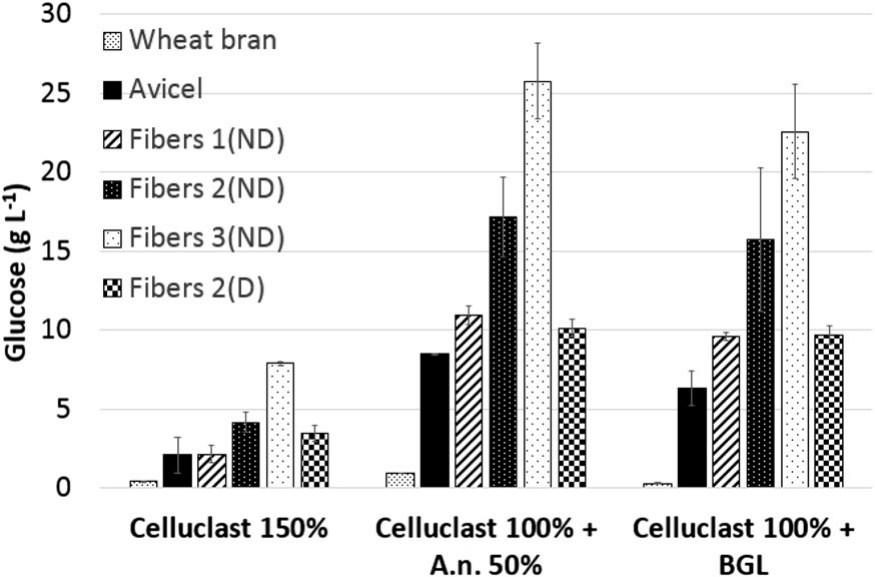
Glucose concentration measured after 24 h hydrolysis at 50 °C of wheat bran, Avicel, Fibers 1(ND), Fibers 2(ND), Fibers 3(ND) and Fibers 2(D). Mean values and standard deviations of two replicates are presented

The overall hydrolysis yield was determined in terms of the total reducing sugars, using the DNS assay (Figs. 1, 2), but this method does not provide any information on the type of sugars, and is therefore more useful for screening purposes. The glucose yield was estimated more precisely using HPAEC-PAD (Fig. 3). The lowest glucose yield was measured during the hydrolysis of wheat bran, and no difference was observed with and without the addition of BGL. Using Celluclast with *A. niger* supernatant resulted in a 3.2 times higher release of glucose from wheat bran than when using Celluclast alone (Fig. 3). Supplementation of Celluclast with *A. niger* supernatant or BGL resulted in 3–5 times higher saccharification than when Celluclast was used alone. However, the glucose yield was higher from all the cellulose-rich substrates when Celluclast was supplemented with *A. niger* supernatant, compared to BGL (Fig. 3).

**In conclusion,** *A. niger* supernatant contains a set of enzymes that significantly improves the *T. reesei* enzymatic cocktail Celluclast 1.5L. BGL, accessory activities and oxidative activities could play a crucial role in improving the hydrolysis of cellulose. It would be interesting to evaluate the supplementation of Cellic CTec enzymatic cocktails, which are superior to Celluclast as they contain BGL and AA9 activities which are absent in Celluclast. Further investigations are required to gain a better understanding of ways in which enzyme mixtures can be optimized for the improved enzymatic hydrolysis of recalcitrant biomass.
